# Fabrication of a Flexible Photodetector Based on a Liquid Eutectic Gallium Indium

**DOI:** 10.3390/ma13225210

**Published:** 2020-11-18

**Authors:** Peng Xiao, Hyun-Jong Gwak, Soonmin Seo

**Affiliations:** Department of Bionano Technology, Gachon University, Seongnam, Gyeonggi 13120, Korea; zhongpengxiao@gmail.com (P.X.); rhkrguswhd@naver.com (H.-J.G.)

**Keywords:** eutectic gallium indium, EGaIn, liquid metal, gallium alloy, flexible photodetector, flexible electronics

## Abstract

A fluidic gallium-based liquid metal (LM) is an interesting material for producing flexible and stretchable electronics. A simple and reliable method developed to facilitate the fabrication of a photodetector based on an LM is presented. A large and thin conductive eutectic gallium indium (EGaIn) film can be fabricated with compressed EGaIn microdroplets. A solution of LM microdroplets can be synthesized by ultrasonication after mixing with EGaIn and ethanol and then dried on a PDMS substrate. In this study, a conductive LM film was obtained after pressing with another substrate. The film was sufficiently conductive and stretchable, and its electrical conductivity was 2.2 × 10^6^ S/m. The thin film was patterned by a fiber laser marker, and the minimum line width of the pattern was approximately 20 μm. Using a sticky PDMS film, a Ga_2_O_3_ photo-responsive layer was exfoliated from the fabricated LM film. With the patterned LM electrode and the transparent photo-responsive film, a flexible photodetector was fabricated, which yielded photo-response-current ratios of 30.3%, 14.7%, and 16.1% under 254 nm ultraviolet, 365 nm ultraviolet, and visible light, respectively.

## 1. Introduction

A fluidic gallium-based liquid metal (LM) is an interesting material for flexible and stretchable electronics and has received much attention from researchers owing to its extraordinary electrical conductivity and outstanding mechanical properties [1,2,3,4]. It is known that various materials have been utilized for manufacturing flexible and stretchable electronics [5,6,7]. However, these materials are not flexible and stretchable in the bulk state and need to be treated further. Interestingly, LM has a fluidic nature at room temperature and thus has potential for various applications in stretchable electronics. With the rapid development of artificial and flexible applications and systems, such as flexible and wearable electronics [8,9], electronic skins [10,11,12], sensors [13,14], and energy harvesting and storage devices [15,16,17], LMs can be utilized for various applications in these fields. In particular, gallium-based LMs, such as eutectic gallium indium (EGaIn, Ga/In 85.8%/14.2%), have been intensively investigated in recent years because their toxicity is lower than that of mercury. For instance, gallium-based LMs can be used as high-elasticity droplets [18], self-powered liquid metal machines [12], conductive traces for circuit boards [19,20], soft electrodes for plasma [21], and reconfigurable antennas [22,23].

The patterning of LM film is another strategy for the fabrication of wearable, flexible, and stretchable devices. In contrast to other solid metals, manipulation of LM is difficult because of its high surface tension in the fluidic state and quick oxidation in air. To overcome this, various patterning methods for gallium-based LM have been developed. LM electrodes with patterned structures have been developed by many facile and cheap printing methods, including 3D printing [24], direct printing [25], inkjet printing [26], stencil printing [27], photolithography [28], masked deposition [29], microcontact printing [30], laser patterning [31] and dielectrophoresis [32,33]. One of these methods, laser patterning, can be used with various materials and is a fast and simple method for fabricating devices [34,35]. Therefore, we tried to fabricate the desired LM patterns by the laser ablation method. It is expected that a thin LM film can be rapidly patterned by a fiber laser marker without fatal damage to the polydimethylsiloxane (PDMS) substrate because only metals can absorb energy at a wavelength of 1064 nm, while PDMS cannot.

In addition to the fabrication of conductive LM film, another main area of this work is the utilization of a newly formed metal oxide layer of LMs during the process. Most LMs based on gallium alloys are rapidly oxidized in contact with oxygen and form an ultrathin metal oxide layer by a self-limiting reaction [2,4,36]. It is known that a transparent Ga_2_O_3_ film is used as a photo-responsive film to measure low-density ultraviolet (254 nm and 365 nm) and visible light [37,38]. Furthermore, it has been reported that the oxidized layer could be exfoliated from the LMs with adhesive materials that are used as 2D materials for the semiconducting layer [39]. Thus, it is considered that the newly formed Ga_2_O_3_ film in this work can be separated with an adhesive material, and this layer would show photo-responsive performance.

In this work, we introduce a simple and reliable method to fabricate a flexible and transparent photodetector based on an LM. A large and thin conductive EGaIn film can be fabricated with compressed EGaIn microdroplets. The LM film is sufficiently conductive and can be rapidly patterned by laser ablation. In addition, a photo-responsive gallium oxide layer can also be separated with an adhesive PDMS substrate from a conductive LM film. A flexible and transparent photodetector was fabricated by combining the patterned LM electrode and the separated Ga_2_O_3_ film.

## 2. Materials and Methods

### 2.1. Fabrication of Liquid Metal (LM) Microdroplets

**Figure 1:**
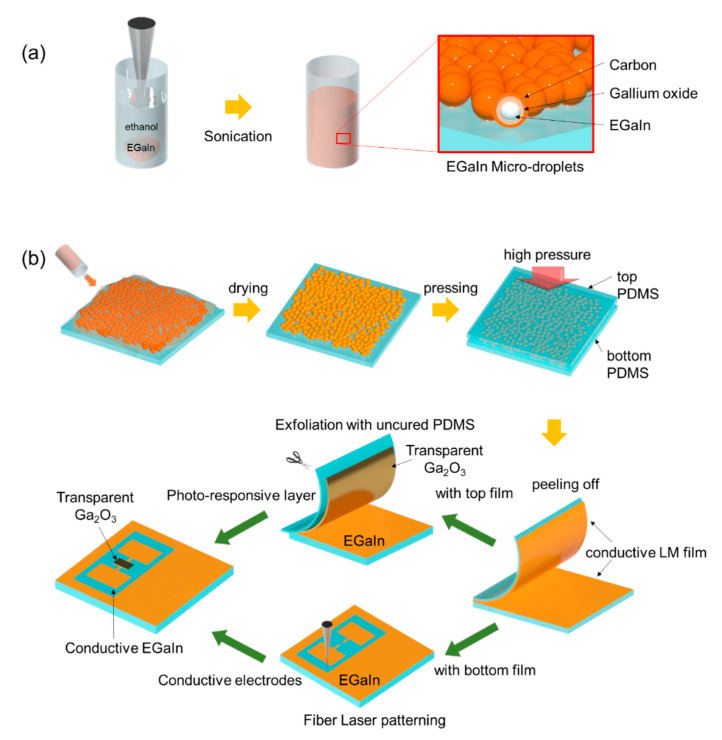
(**a**) Preparation process of EGaIn microdroplets; (**b**) Illustration of the fabrication process of a flexible and transparent photodetector.

EGaIn (75.5 wt% Ga and 24.5 wt% In, Sigma-Aldrich, St. Louis, MO, USA) was used to prepare the microdroplets. It consists of Ga and In, and its melting point is 15.7 °C. EGaIn (500 mg) was placed in a 20 mL vial and was filled with ethanol (94.5%, Daejung, Korea). Hereafter, the vial was sonicated using an ultrasonic cleaner (80 W, 40 KHz) for 30 min. After ultrasonication, a suspension of LM microdroplets (<10 μm) was formed, as shown in Figure 1a.

### 2.2. Preparation of Fully and Incompletely Cured PDMS Substrates

The fully-cured PDMS substrate was prepared by the following process: A PDMS (Dow Corning, Sylgard 184 A/B) mixture with a monomer and a curing agent (at a ratio of 10:1) was prepared and poured onto a flat Petri dish (SPL, Gyeonggi, Korea). The bubbles arising from the vacuum chamber were removed after 1 h, post which it was cured in a convection oven for another 1 h at 80 °C. For an incompletely-cured PDMS substrate, a PDMS mixture with a monomer and a curing agent (at a ratio of 11:1) was used. The mixture was poured on a flat Petri dish, and the height of the incompletely-cured PDMS substrate was 1 mm. It was then cured in an oven for 15 min at 80 °C after removing the bubbles.

### 2.3. Fabrication of Thin Conductive LM Film with Microdroplet Suspension

A suspension of LM microdroplets formed by ultrasonication was dropped on a flat, fully-cured PDMS substrate and dried at room temperature for 24 h to avoid formation of cracks by rapid solvent evaporation. Subsequently, another flat, fully-cured PDMS substrate was placed onto the dried LM film, which was then pressed by a hydraulic press at 15 MPa for 1 s. After removing the pressure, the upper PDMS substrate was peeled off from the bottom substrate. Finally, thin conductive LM films were left on both the upper and bottom PDMS substrates.

### 2.4. Laser-Engraved Conductive Patterns and Circuits

The thin liquid metal (LM) film was patterned by a fiber laser marker (50 W, Dongil laser technology, Gyeonggi, Korea). The desired circuits and electrodes were fabricated by a subtractive method at a resolution of 20 μm. The scanning speed of the fiber laser marker was 600 mm/s, and the power intensity of the laser was 1% of its maximum power. A pattern with an area of 4 cm^2^ could be engraved within 5 s by a fiber laser marker.

### 2.5. Fabrication of a Photodetector Based on Oxidized LM Film

An incompletely-cured thin PDMS film was placed slightly on the surface of a gallium-based conductive thin film for conformal contact. The gallium oxide (<10 nm) film on the LM was attached to the incompletely-cured PDMS film, and it was exfoliated from the conductive film after the peeling off process. After cutting down a part of the transparent gallium oxide film on the PDMS film, it was then placed onto the laser-patterned EGaIn electrodes. Finally, the photodetector was fabricated with a transparent gallium oxide film and patterned conductive EGaIn electrodes.

### 2.6. Characterization

A semiconductor characterization system (Keithley 4200, Beaverton, OR, USA) was used to analyze the electrical properties of the conductive electrodes. Bending and stretching tests were also performed. Photo-detective tests under the irradiation of an ultraviolet (UV) lamp (8 W, Vilber Lourmat, Marne La-Vallee, France) were also carried out at various wavelengths. The tests were also done under a tungsten–halogen lamp (FOK-100 W, Fiber Optic Korea, Cheonan, Korea). Atomic force microscopy (AFM) and scanning electron microscopy (SEM) images were obtained using a multimode AFM (Nanoscope IIIa, Digital Instruments, Bresso, Italy) and FE-SEM (JSM-7500F, Jeol, Tokyo, Japan), respectively.

## 3. Results

LM based on gallium alloys (EGaIn) was used for the device. In Figure 1a, the preparation process of EGaIn microdroplets is shown. A suspension of LM microdroplets was formed by ultrasonication in ethanol. The inner core of the droplet is EGaIn and is surrounded by gallium oxide with an outer carbon shell [40,41,42]. In Figure 1b, a schematic illustration of the entire process is shown. The solution was dropped on a flat, fully-cured PDMS substrate and dried at room temperature for 24 h to avoid the formation of cracks by rapid solvent evaporation during the drying process.

**Figure 2:**
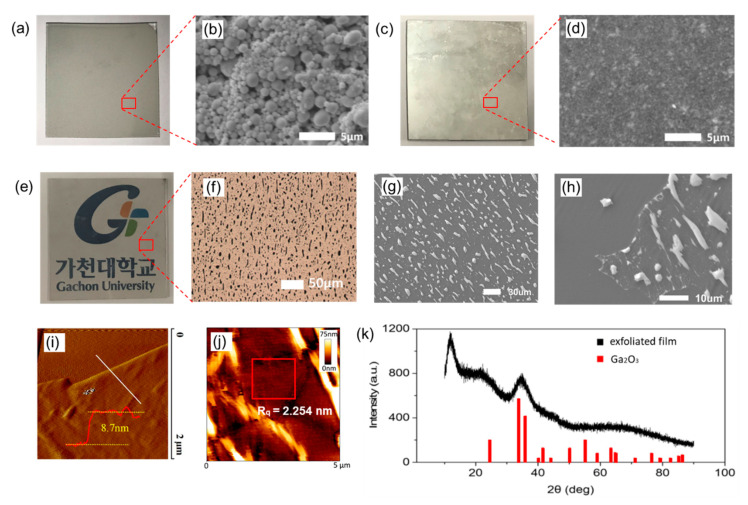
(**a**) Liquid metal microdroplets distributed on a PDMS substrate; (**b**) SEM image of the liquid metal (LM) microdroplets; (**c**) Image of a thin conductive LM film after peeling off process; (**d**) SEM image of the continuous conductive LM film on a PDMS substrate; (**e**) The transparent gallium oxide film attached on a PDMS after peeling off from the LM film; (**f**) Magnified optical image; (**g**) SEM image of the transparent film of (**e**); (**h**) a piece of the thin tore transparent film on a PDMS substrate; (**i**) The thickness and (**j**) the roughness of transparent gallium oxide film (boxed area) by AFM; (**k**) XRD pattern of the exfoliated film on a PDMS substrate.

LM droplets by ultrasonication were distributed on the PDMS substrate after solvent evaporation, as shown in Figure 2a. EGaIn microdroplets synthesized by ultrasonication were distributed uniformly on the PDMS substrate, and the size of the droplets was smaller than 3 μm, as shown in Figure 2b. Round droplets were observed, and the droplets were wrapped with oxidized gallium material. The film with stacked LM droplets itself is not conductive because it is difficult for an electron to move a long distance through the nonconductive oxidized layers from one droplet to another. The advantage of using the droplets formed by ultrasonication is that it is possible to fabricate a thinner and more uniform LM film when the droplets are used. With bulk EGaIn, it is difficult to create a thin film because the high surface tension of a bulk LM makes it difficult to manage the LM.

Subsequently, another flat, fully-cured PDMS substrate was placed onto the dried microdroplet film, which was then pressed by a hydraulic press at a pressure of 15 MPa. The droplets were squashed out and connected to each other by pressure after breaking the oxidized layers of the droplets. The oxidized parts remained in the film inside. However, the connected droplets formed a large conductive thin film, as shown in Figure 2c,d. The amount of oxidized material is much lower than that of the conductive part, and the film is sufficiently conductive for use in electric devices. The top PDMS substrate on the sandwiched LM film was then peeled off. After that, half of the LM remained on the bottom substrate, and another half was transferred onto the top substrate. As a result of the peeling process, two conductive LM sheets were obtained on the top and bottom PDMS substrates. The LM film was formed on the flat PDMS substrate over the entire area. It is difficult to fabricate a very thin LM film from bulk LM because of its high surface tension. However, this difficulty has been overcome by using LM microdroplets. In this work, one of the LM-coated sheets (bottom) was used as a conductive electrode, and another (top) was used for utilizing a photo-responsive layer.

Moreover, an extremely thin oxidized layer could be separated from the LM films by peeling off with a sticky PDMS film. The incompletely-cured PDMS film with strong adhesion was designed to peel off the oxidized layer from the surface of the LM thin film for large-area fabrication. The incompletely-cured PDMS film was placed and covered on the surface of the LM thin film, and it was brought into contact with the oxidized layer of the LM, forming a conformal contact. Hereafter, the transparent oxidized layer was sliced out from the LM film, at the time of peeling off the uncured PDMS film. The optical image of the separated gallium oxide film from the LM film is shown in Figure 2e. The film is very thin and transparent. As shown in Figure 2f,g, small LM spots (<10 μm) remained on the transparent film. However, all the spots were surrounded by gallium oxide film and isolated from each other. Thus, the separated gallium oxide film is laterally nonconductive. The transparent nonconductive film was analyzed by grazing incidence X-ray diffraction (GIXRD). The oxidized sample was prepared on a PDMS substrate, and the graph obtained by GIXRD is shown in Figure 2k. The baseline was similar to reported GIXRD data of bare PDMS [43], and a broad peak was observed around 35°. It is known that GIXRD peaks are also observed at 34° and 36° for a gallium oxide material. Thus, it is concluded that the transparent film is gallium oxide, since the peaks of gallium oxide appear on the graph, and the material was also responsive to UV light in this work. Finally, the separated transparent film was used for fabricating a photodetector because the oxidized thin film is a material mainly based on gallium oxide and it shows high photo-detective property, as reported previously [37,38]. The separated oxidized film on a PDMS substrate could be easily cut using a scissor. Then, it was placed on the substrate between the cathode and anode to fabricate a photodetector. After placing the gallium oxide on the PDMS film between the electrodes, the photodetector with transparent film and flexible LM electrodes was completed, as shown in Figure 1b.

**Figure 3:**
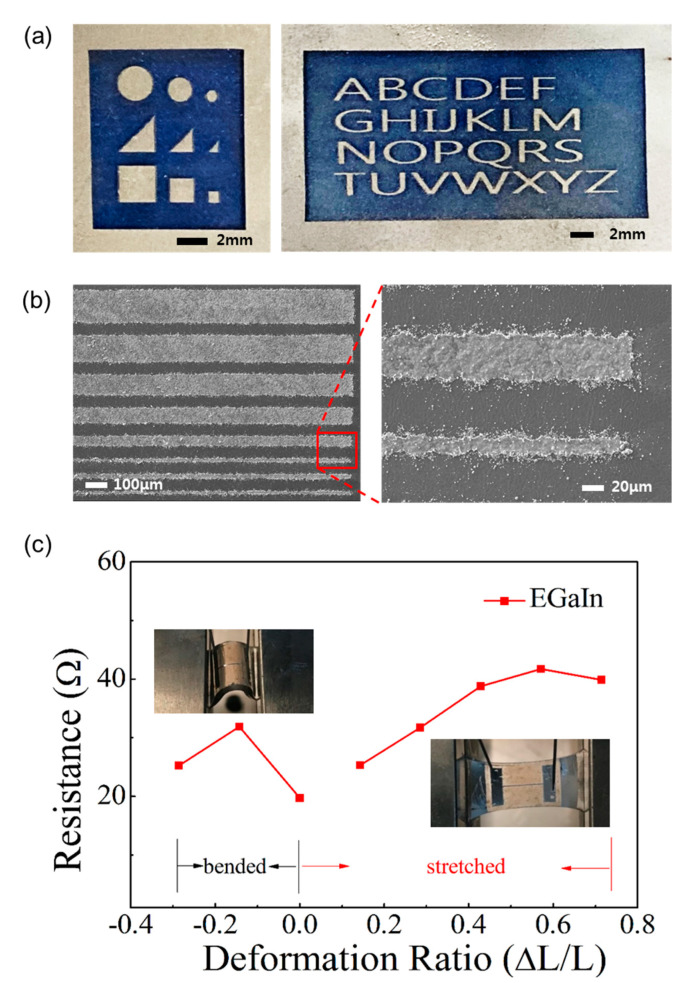
(**a**) Images of a conductive EGaIn film patterned by a fiber laser marker; (**b**) SEM images of conductive EGaIn film patterned by a fiber laser marker, showing a maximum resolution of the pattern of approximately 20 μm; (**c**) Resistance of the EGaln electrode under bending and stretching.

In addition, to fabricate a desirable pattern for a flexible and stretchable device, a fiber laser marker (λ ~ 1064 nm) was used for designing the patterns on a conductive LM film. The fabricated conductive films are shown in Figure 3a. A laser engraving method is an efficient way to establish flexible electrodes and patterns for devices. The SEM images in Figure 3b show the pattern with various sizes based on EGaIn by a fiber laser marker. The advantages of using a fiber laser marker for the patterning process are fine pattern resolution and less damage to transparent substrates, such as PDMS or glass, during the patterning process. In fact, buckling of the PDMS substrate due to heat was observed when LM was blazed out. The method can establish a sub-100 μm pattern, and the minimum line was approximately ~20 μm in the experiment. Furthermore, better resolution can be achieved using the laser blazing method if highly qualified equipment is used for patterning [44]. In a previous report, a CO_2_ laser, not a fiber laser, was used for cutting the LM electrode inside the PDMS substrate [31]. Actually, the CO_2_ laser blazed out the PDMS, and not the LM, in the experiment. In this work, different mechanisms were used. It is known that metal substrates absorb only a small amount of energy from the CO_2_ laser, and most of the energy from the CO_2_ laser is reflected [45]. In contrast, a metal can absorb the energy of a fiber laser. Thus, the fiber laser is suitable for patterning thin LM films. It could easily remove a thin LM film (<1 μm) quickly. Complex patterns with a resolution below 100 μm (~20 μm) could be made using a fiber laser in this work.

After peeling off the top PDMS substrate, half of the LM remained on the bottom substrate and another half was transferred to the top substrate, as shown in Figure 1b. As a result of the peeling process, two conductive LM sheets were obtained. Resistance measurement during the stretching test was also performed with an LM electrode (5 mm long and 80 μm wide). It was measured by a semiconductor parameter analyzer, and the characteristic performances of the flexible electrodes are shown in Figure 3c. Resistance of the EGaIn film is between 19.7 Ω and 41.7 Ω; this increases slightly when the film is stretched to 170% of its initial length. The electrical conductivity of the EGaIn film in this work was 2.2 × 10^6^ S/m. The value is approximately two-thirds of its known value (pure EGaIn, ~3.4 × 10^6^ S/m) and was measured using a resistivity meter (Loresta-GX MCP-T700, Mitsubishi Chemical Analytech, Yamato, Japan) with a four-pin probe to overcome the effect of contact resistance. According to the results, the film is sufficiently conductive to be used as an electrode in the circuit. Here, one of the LM sheets was used as a conductive electrode and the other was used for a photo-responsive layer. The thickness of the LM films measured by AFM was approximately 600 nm. It is known that it is difficult to fabricate a very thin LM film because of its high surface tension. However, we could overcome this difficulty by using LM droplets and making a thin (< 1 μm) LM film on the substrate.

## 4. Discussion

### 4.1. Fabrication of the Photodetector

In Figure 2b, a conductive LM thin film and a transparent gallium oxide film that is separated from the LM film are shown. All films were fabricated on a large PDMS substrate (5 × 5 cm). As shown in Figure 2d, the morphology of the conductive LM film was not smooth because LM was immediately oxidized and solidified when exposed to air after the peeling-off process. The thickness of the conductive LM film measured by AFM was approximately 600 nm. To measure the thickness of the exfoliated gallium oxide layer, the transparent film on the PDMS substrate was transferred onto a silicon substrate. The measured thickness of the exfoliated metal oxide film by AFM was 8.7 nm, as shown in Figure 2i, and the surface roughness (RMS roughness) of the exfoliated 2D Ga_2_O_3_ layer for a flat area was 2.254 nm, as shown in Figure 2j. It is assumed that the measured value of the gallium oxide layer is thicker than the known value (~3 nm) because there is further oxidation during the separation process. In this work, the separated transparent film was used as an active layer in a photodetector because the oxidized film based on the gallium oxide is highly photo-detective.

### 4.2. Device Characterization

**Figure 4:**
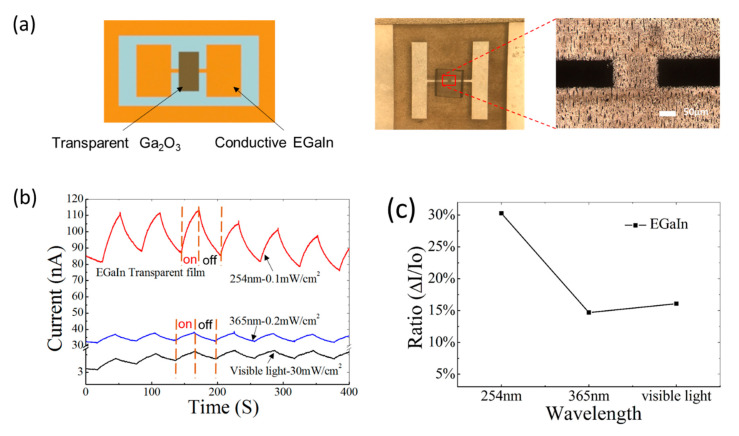
(**a**) Images of the photodetector combined with the conductive electrode and photo-responsive film; (**b**) Time-dependent photo-response curves and (**c**) photo-response-current ratios of the photodetector under illumination with ultraviolet light (λ~254 nm and 365 nm) and visible light. The bias voltage was 0.1 V, and the on/off time of lights was 30 s/30 s.

A photo-sensitive device was fabricated with flexible electrodes and a transparent gallium oxide film based on LM(EGaIn), as shown in Figure 4a. The black rectangular areas on the right side of the figure are the LM electrodes patterned by a fiber laser, and the transparent area is a gallium oxide film beneath the PDMS substrate. The center image in Figure 4a shows the bird’s-eye view of the full structure of the device.

It is known that Ga_2_O_3_ has a wide bandgap (4.5~4.9 eV) at room temperature [46,47]. As a result, the device is used to measure ultraviolet and visible light as a high-range photodetector. As shown in Figure 4b, the characteristics show an obvious photo-responsive performance under periodic illumination. Photo-responsive tests were also performed with light of three different wavelengths. The devices were illuminated by light periodically at intervals of 30 s with three wavelengths: 254 nm, 365 nm, and visible light. The rise/decay times of the device under illumination of 254 nm, 365 nm, and visible light were 28.2 s/26.7 s, 18.3 s/21.9 s, and 29.1 s/23.6 s, respectively. The responsivities (R) under illumination of 254 nm, 365 nm, and the visible light at 1 V were 2.8 × 10^−2^ A/W, 3.3 × 10^−3^ A/W, and 2.6 × 10^−6^ A/W, respectively. As shown in Figure 4c, the device based on EGaIn shows a photo-response-current ratio (∆I/I_0_) of 30.3% under 254 nm ultraviolet light with an intensity of 0.1 mW/cm^2^. It also shows photo-response–current ratios of approximately 14.7% and 16.1% under the illumination of a 365 nm ultraviolet and an ordinary visible light, respectively.

## 5. Conclusions

This work describes a new type of flexible photodetector based on a liquid gallium alloy. A simple and reliable method was introduced to fabricate a flexible and transparent photodetector based on LMs. The photodetector was fabricated with a material, EGaIn. Both a conductive electrode and a photo-responsive layer could be obtained from the material and fabricated on PDMS substrates. The fabrication process of a conductive film based on LM microdroplets is an efficient method to fabricate a large-area (5 × 5 cm), flexible, and stretchable LM film. The laser ablation method was also used to fabricate flexible and stretchable electrodes, and the width of the patterned electrodes could be controlled at a level of 20 μm. A photo-responsive layer (~8.7 nm) was exfoliated with an incompletely-cured PDMS by peeling off from the surface of the oxidized LM film. Finally, the photodetector could be made by combining the patterned electrodes and the photo-responsive film. It shows 30.3%, 14.7%, and 16.1% of the photo-response–current ratio under wavelengths of 254 nm and 365 nm in the ultraviolet band, and ordinary visible light, respectively.

The key contribution of this method is that a photosensitive device was fabricated with one material. The semiconducting active layer was exfoliated from conductive materials, and both layers were used in the same device. The laser ablation method shows high performance of controllable, accurate, and efficient patterning. It is expected that the results with LM and various techniques in this work will contribute to advances in the fields of flexible and stretchable sensors.
